# Survival outcomes associated with antidepressant use in glioblastoma: a cohort study

**DOI:** 10.1007/s11060-025-05288-3

**Published:** 2025-10-27

**Authors:** Yifei Sun, Mohammad Hamo, Travis Atchley, James M. Markert, Burt Nabors, Dagoberto Estevez-Ordonez

**Affiliations:** 1 Marnix E. Heersink School of Medicine, University of Alabama at Birmingham, Birmingham, AL USA; 2 Department of Neurosurgery, University of Alabama at Birmingham, Birmingham, AL USA; 3 O’Neal Comprehensive Cancer Center, University of Alabama at Birmingham, Birmingham, AL USA; 4 Division of Neuro-Oncology, University of Alabama at Birmingham, Birmingham, AL USA; 5 Department of Neurological Surgery, University of Miami & Jackson Health System, 1095 NW 14th Terrace, 2nd FL, Miami, FL 33136 USA

**Keywords:** Antidepressants, Glioblastoma, Survival

## Abstract

**Purpose:**

Glioblastoma is the most common primary brain malignancy and carries significant mortality. Preclinical studies have highlighted the efficacy of antidepressant therapy in inhibiting glioblastoma progression; however, real-world evidence remains conflicting. We sought to investigate the impact of different commonly utilized antidepressant therapies on survival in patients with glioblastoma.

**Methods:**

In total, 1464 consecutive patients with glioblastoma treated at a single institution from 2008 to 2023 were included for analysis. Multivariate cox regression analysis with antidepressant usage modeled as a time varying covariate was used to assess the effect of antidepressants while controlling for a priori selected clinical variables with known relevance to survival.

**Results:**

The median age at diagnosis was 62 (IQR 52–70) years with a median overall survival of 13.8 months. Of the cohort, 44% utilized antidepressants after diagnosis, with SSRIs as the most common class utilized (26%). The median duration of any antidepressant therapy was 111 (IQR 9-303) days. In a time varying, multivariate cox regression, usage of SSRIs (HR 1.4, 95%CI 1.21–1.62), SNRIs (HR 1.33, 95%CI 1.03–1.72), serotonin modulators (HR 1.61, 95%CI 1.40–1.86), and atypical antidepressants (HR 1.7, 95%CI 1.28–2.26) were associated with worse survival. Amongst SSRIs, only escitalopram (HR 1.33, 95%CI 1.10–1.60) and citalopram (HR 1.31, 95%CI 1.01–1.70) were associated with worse survival.

**Conclusions:**

Antidepressant therapy is associated with worse survival in patients with glioblastoma after adjusting for known factors with relevance to survival. Clinicians should consider the risks and benefits of prescribing antidepressants in patients with glioblastoma. Further evidence is necessary to better understand the impact of antidepressant therapy in glioblastoma survival.

**Supplementary Information:**

The online version contains supplementary material available at 10.1007/s11060-025-05288-3.

## Introduction

Glioblastoma is the most common primary central nervous system malignancy in adults, accounting for nearly half of primary brain tumors [1]. On the current standard of care of maximal safe surgical resection followed by radiation therapy and adjuvant chemotherapy, survival remains poor. Despite recent improvements in therapy delivery and innovations in treatment regimens, glioblastoma carries a poor prognosis, with median survival of around 15 months [1, 2]. Thus, it remains of high interest to further develop novel therapies to better patient survival.

Disproportionally high rates of depression is a well-known comorbidity of glioblastoma, and is associated with poor patient outcomes [3–5]. Depression may occur in nearly 40% of patients with glioblastoma, and antidepressant therapy is frequently prescribed for management of these symptoms [4]. The potential ways in which antidepressant therapy my improve glioblastoma outcomes are many. Improvement of patient’s depressive symptoms may improve function, leading to decreased deterioration, increased adherence to treatment regimes, and improved activities of daily living (ADL) [6, 7]. Many pre-clinical studies highlight the interplay between antidepressant therapy and glioblastoma signaling pathways. Several studies have demonstrated the ability of antidepressants to inhibit invasiveness and increase autophagy [8, 9]. Some studies have demonstrated the ability of antidepressant medications to suppress transcription factors associated with glioblastoma progression in vitro [10]. Still others have demonstrated strong anti-glioblastoma effects in mice models as well [11–13].

However, the effect of antidepressant therapy on glioblastoma survival is inconclusive in literature. Analysis by Caudill et al. [14] found SSRI therapy to be associated with improved survival, while Seliger et al. [15] found antidepressant use to be associated with worse survival. In analysis by Edstrom et al. [16] using a multicenter registry, SSRI therapy and non-SSRI antidepressant therapy was found to be associated with worsened survival, while analysis by Otto-Meyer et al. [17] found non-significant results. Recent meta-analysis exploring this topic suggest inconclusive findings, limited studies, and high degrees of heterogeneity [18, 19].

The effects of antidepressant therapy on glioblastoma survival remains unclear, and the effect of specific classes of antidepressants have not been explored. Furthermore, the association of antidepressants and glioblastoma has not been explored while taking into account socioeconomic and molecular factors associated with survival [20, 21]. We sought to characterize the independent effect of antidepressants on glioblastoma survival while accounting for molecular and socioeconomic status. We additionally sought to understand the differential impact of different antidepressant classes on glioblastoma survival.

## Methods

This study was designed as a single center retrospective review with approval from the institutional review board (IRB-300005353). This manuscript was written in compliance with STROBE (Strengthening the Reporting of Observation Studies in Epidemiology) [22].

### Participants and data collection

We retrospectively identified all adult patients with histopathological confirmed glioblastoma who were treated at our institution between January 2008 and December 2023 with complete medication records. We reviewed the electronic medical record (EMR) for variables on patient demographics, treatment characteristics, and medication records. Patient consent was not sought due to the retrospective nature of this study.

### Defining variables

Variables were defined a priori with advice from the senior authors (DEO, JM, BN). The study variables included were age at diagnosis categorized according to standard groups (< 45, 45–54, 55–64, 65–74, and ≥ 75), race (white, African American, and other), gender (Male or Female), and insurance status, which was categorized as private, public (Medicare, Medicaid, Tricare), or indigent/self-pay, extent of resection, IDH mutation status, MGMT methylation status, treatment history such as history of chemotherapy and radiotherapy [23]. Patient addresses were extracted and geocoded and linked to federal information processing (FIPS) codes. Neighborhood deprivation, captured by Area Deprivation Index (ADI), was retrieved from the Neighborhood Atlas dataset produced by the Center for Health Disparities Research at the University of Wisconsin School of Medicine and Public Health, with higher ADI indicating a higher level of socioeconomic disparity [24]. High ADI was defined as being in the top quartile of disadvantage nationally.

Rural urban communicating area (RUCA) codes were extracted and categorized in accordance with the Economic Research Service (ERS) of the United States Department of Agriculture and divided into the 4 main categories of metropolitan, micropolitan, small town, and rural [25].

Patient medication records were reviewed for antidepressant usage after glioblastoma diagnosis. Usage was counted as date first prescribed to the end date on the prescription or censoring, whichever came first. Antidepressants were defined into 5 categories: selective serotonin reuptake inhibitors (SSRIs), serotonin/norepinephrine reuptake inhibitors (SNRIs), serotonin modulators (SMODs), tricyclic antidepressants (TCAs), and atypical antidepressants. The most common drugs for each category were selected for inclusion. Specific medications chosen for inclusion can be found in the supplementary content (Supplementary Digital Content, Supplementary Methods).

### Statistical analysis

Categorical, binary, and ordinal variables were summarized as counts and percentages, while continuous variables were summarized as the median and interquartile range (IQR). Univariable comparison analysis was performed via utilizing the one-way analysis of variance (ANOVA), log-rank test, Pearson’s chi-squared test, Wilcoxon rank sum test, or Fisher’s exact test. Simon-Makuch plots with Mantel-Byar method were utilized to visualize unadjusted time-varying survival curves [26, 27].

To assess the independent effect of various antidepressants on survival, multivariate cox regression models were utilized with antidepressant usage modeled as a time varying covariate to assess the association of various antidepressant therapies with glioblastoma overall survival (OS) while controlling for age, insurance status, race, neighborhood disadvantage, MGMT methylation status, IDH mutation status, treatment with chemotherapy, treatment with radiotherapy, extent of resection, RUCA code status, and comorbid depression and/or anxiety. Utilization of a time varying covariate model allows the model to account for changes in exposure status over the period of follow-up, addressing immortal time bias and allowing for a more appropriate comparison of survival effects of the exposure of interest [28]. There was a high degree of missing values for MGMT methylation (39%) and IDH mutation (33%) status. Because most of the missing values were before 2016, we assumed that the data was missing at random (MAR) due to inconsistent biomolecular marker testing before the release of the 2016 WHO Guidelines on Tumors of the Central Nervous System [29–31]. We performed multiple imputations using the *missForest* random forest classifier, which resulted in an out of box (OOB) of 2%, demonstrating high imputation accuracy (Supplementary Digital Content, Figure S1).

To conduct sensitivity analysis to demonstrate the robustness of our findings, we replicated the cox regression models using complete case analysis, and in a cohort of patients with comorbid or pre-existing depression and/or anxiety. Statistical significance was set at α = 0.05, and all tests for significance were two-sided. All statistical analyses were performed using R (version 4.3.1, R Foundation for Statistical Computing, Vienna, Austria) [32].

## Results

### Patient characteristics and demographics

In total, 1464 patients were included for analysis. The median age at diagnosis was 62 [Interquartile range (IQR) 52–70], with 648 (44%) being female. Of these patients 155 (11%) were African American, and 49% had private insurance. Of these patients, 671 (46%) underwent gross total resection (GTR), 1219 (83%) had received chemotherapy, and 1235 (84%) had received radiation therapy. Of the cohort, 44% of patients had some form of antidepressant therapy, with the most common being SSRI therapy (26%) followed by serotonin modulator therapy (22%) and SNRI therapy (5.9%). Further details on patient characteristics can be found in Table 1.

**Table 1:** Patient characteristics and demographics

Characteristic	*N* = 1,464^1^
Age (years)	62 (52, 70)
Sex	
Female	648 (44%)
Male	816 (56%)
Race	
White	1,224 (84%)
Black	155 (11%)
Other	85 (5.8%)
Insurance type	
Private	712 (49%)
Public	701 (48%)
Self-Pay/Indigent	51 (3.5%)
RUCA code	
Metropolitan	1,062 (73%)
Micropolitan	225 (15%)
Rural	51 (3.5%)
Small Town	126 (8.6%)
ADI Rank	66 (46, 84)
Vital Status at Last Follow-up	
Alive	249 (17%)
Deceased	1,215 (83%)
IDH Status	
IDH-Mut	92 (9.4%)
IDH-WT	890 (91%)
Unknown	482
MGMT status	
Methylated	344 (39%)
Unmethylated	544 (61%)
Unknown	576
Chemotherapy	1,219 (83%)
Radiotherapy	1,235 (84%)
Extent of Resection	
Biopsy	430 (29%)
Gross Total Resection	671 (46%)
Partial Resection	363 (25%)
Comorbid Depression/Anxiety	432 (30%)
Any Antidepressants	647 (44%)
SSRI	377 (26%)
Serotonin Modulators	316 (22%)
SNRI	87 (5.9%)
Atypical Antidepressants	69 (4.7%)
TCAs	49 (3.3%)
MAOI	3 (0.2%)

^1^ Median (Q1, Q3); n (%), SSRI: Selective Serotonin Receptor; SNRI: Serotonin/Norepinephrine Reuptake Inhibitors; TCA: Tricyclic antidepressants; MAOI: Mono-amine oxidase inhibitors; RUCA: Rural urban communicating area; ADI: Area Deprivation Index

### Univariable comparison

Patients who received antidepressant therapy were younger (61 vs. 63 years, *p* = .016), more likely to be female (48% vs. 41%, *p* = .009), more likely to be white (88% vs. 80%, *p* < .001), more likely to have received chemotherapy (86% vs. 81%, *p* = .01), radiotherapy (87% vs. 82%, *p* = .039), and more likely to had undergone gross total resection (49% vs. 43%, *p* < .001) (Table 2).

**Table 2:** Comparison by antidepressant therapy

Characteristic	Had Antidepressant Therapy		*p*-value^2^
	No	Yes	
	*N* = 817^*1*^	*N* = 647^*1*^	
Age	63 (53, 71)	61 (51, 69)	0.016
Sex			0.009
Female	337 (41%)	311 (48%)	
Male	480 (59%)	336 (52%)	
Race			< 0.001
White	657 (80%)	567 (88%)	
Black	91 (11%)	64 (9.9%)	
Other	69 (8.4%)	16 (2.5%)	
RUCA code			0.7
Metropolitan	593 (73%)	469 (72%)	
Micropolitan	130 (16%)	95 (15%)	
Rural	29 (3.5%)	22 (3.4%)	
Small Town	65 (8.0%)	61 (9.4%)	
Area Deprivation Index	67 (47, 84)	66 (44, 84)	0.2
IDH Status			0.3
IDH-Mut	52 (10%)	40 (8.4%)	
IDH-WT	451 (90%)	439 (92%)	
MGMT Status			0.7
Methylated	176 (39%)	168 (38%)	
Unmethylated	272 (61%)	272 (62%)	
Chemotherapy	662 (81%)	557 (86%)	0.01
Radiotherapy	674 (82%)	561 (87%)	0.028
Extent of Resection			0.039
Biopsy	261 (32%)	169 (26%)	
Gross Total Resection	355 (43%)	316 (49%)	
Partial Resection	201 (25%)	162 (25%)	
Comorbid Depression or Anxiety	78 (9.5%)	354 (55%)	< 0.001

^*1*^ Median (Q1, Q3); n (%)

^*2*^ Wilcoxon rank sum test; Pearson’s Chi-squared test; Fisher’s exact test, RUCA: Rural urban communicating area; ADI: Area Deprivation Index

### Antidepressant prescribing patterns

**Fig. 1:**
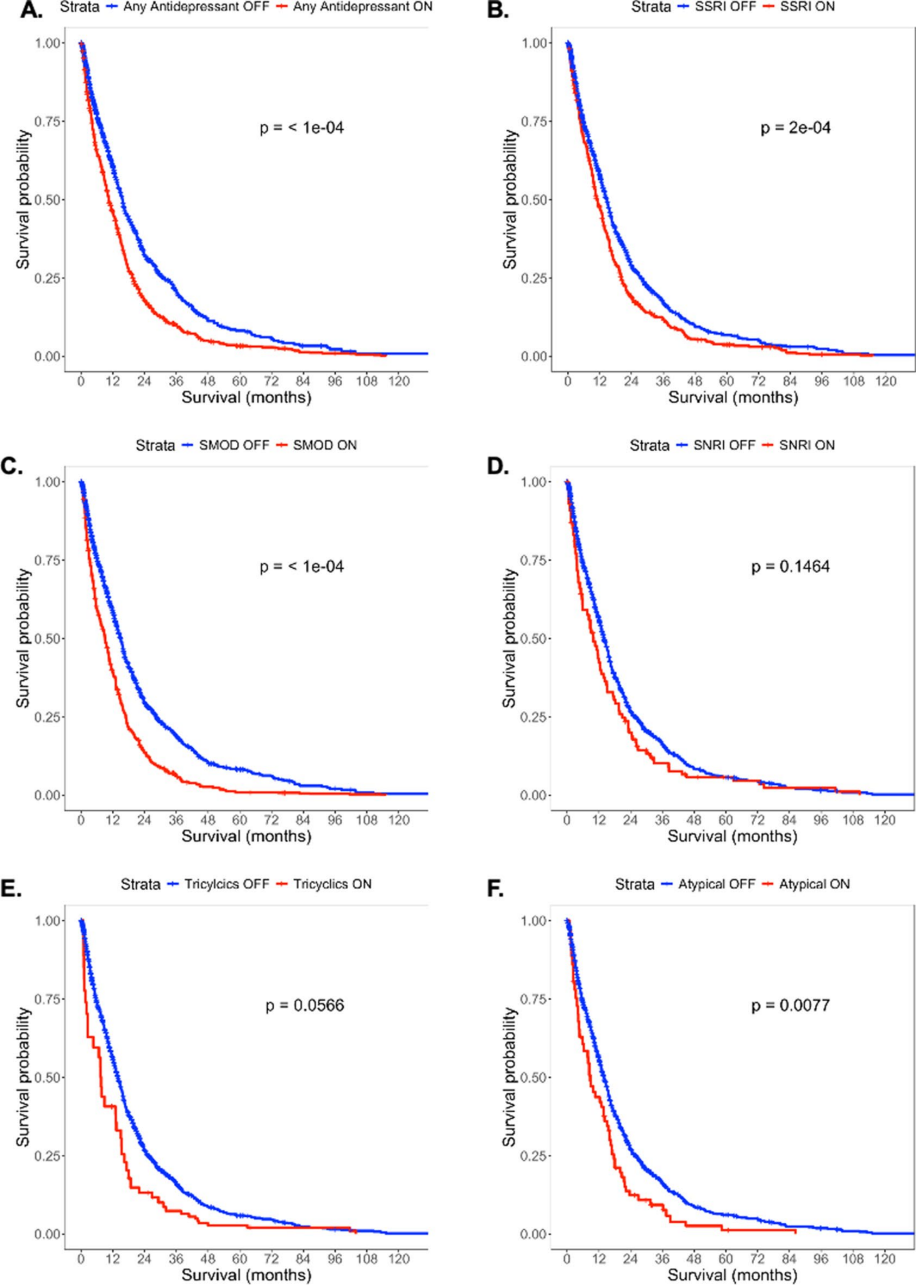
Simon Makuch plots showing unadjusted survival for (**A**) Any antidepressant use, (**B**) SSRI use, (**C**) SMOD use, (**D**) SNRI use, (**E**) Tricyclic use, (**F**) Atypical antidepressant use

The most commonly prescribed category of antidepressants were SSRIs, followed by serotonin modulators and SNRIs (Table 1). The median duration of time on antidepressant therapy amongst the cohort was 111 (IQR 9-303) days. Amongst the SSRIs, the median duration dose was 20 (IQR 10-35.9) days, and escitalopram was the most commonly prescribed, followed by sertraline and citalopram. Of the SNRIs, the median duration was 120 (IQR 13.5-402.5) days and duloxetine was the most commonly prescribed followed by venlafaxine. Of the atypical antidepressants, the median duration was 65 (IQR 14–274) days, with mirtazapine and bupropion as the most prescribed. Of the serotonin modulators, the median duration was 26.5 (IQR 5-193.5) days, and trazodone was the most prescribed. Of the MAOIs, the median duration was 10 (IQR 8-107) days, and rasagiline was the most prescribed (Table 3, Supplementary Digital Content Table S1). Of the TCAs, the median duration was 46 (IQR 4-183) days, with amitriptyline being the most commonly prescribed. Of the cohort, 137 patients had some form of antidepressant polytherapy, with the most common overlap being SSRIs and serotonin modulators, followed by SSRIs and atypical antidepressants (Supplementary Digital Content, Figure S2). Univariate Simon-Makuch plots showing unadjusted survival are shown in Fig. 1.

**Table 3:** Antidepressant usage patterns

Drug Name	*N*	Median duration days	Median Daily dose (IQR) mg
Any Antidepressant	647	111 (9, 303)	
SSRI	377	111 (11, 360)	20 (10, 35.9)
SNRI	87	120 (13.5, 402.5)	60 (37, 75)
Serotonin Modulator	316	26.5 (5, 193.5)	50 (37.5, 69)
TCA	49	46 (4, 183)	30 (25, 50)
MAOI	3	10 (8, 107)	1 (0.75, 3.25)
Atypicals	69	65 (14, 274)	30 (15, 150)

SSRI: Selective Serotonin Receptor; SNRI: Serotonin/Norepinephrine Reuptake Inhibitors; TCA: Tricyclic antidepressants; MAOI: Mono-amine oxidase inhibitors

### Survival analysis

**Fig. 2:**
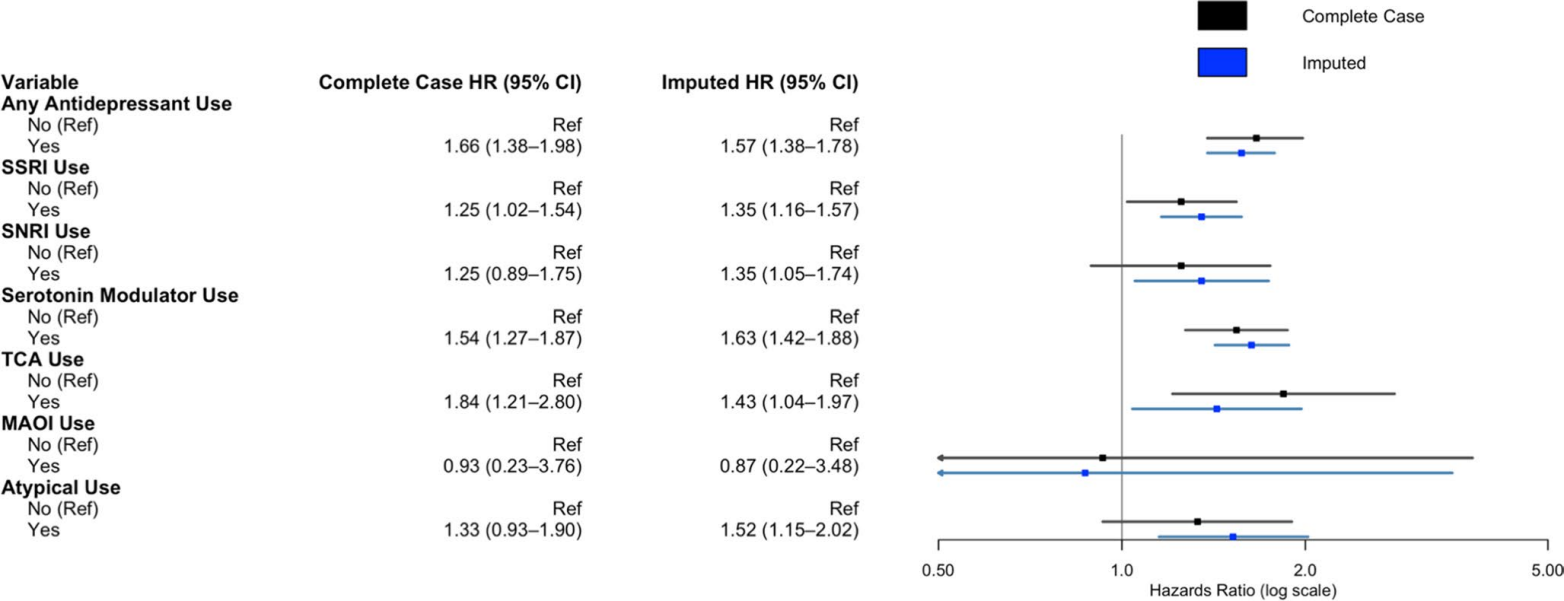
Multivariate cox regression model for impact of antidepressant usage and survival

On multivariate cox regression analysis adjusting for age, comorbid depression or anxiety, insurance payer type, race, neighborhood socioeconomic disadvantage, MGMT methylation status, IDH mutation status, treatment with chemotherapy, treatment with radiotherapy, extent of resection, and rurality, usage of any antidepressant (HR 1.57, 95%CI 1.38–1.78, *p* < .001) was associated with worse survival. In multivariate cox regression controlling for the same cofactors but investigating individual antidepressant classes, SSRI usage (HR 1.35, 95%CI 1.16–1.57, *p* < .001), SNRI usage (HR 1.35, 95%CI 1.05–1.74, *p* < .02), serotonin modulator usage (HR 1.63, 95%CI 1.42–1.88, *p* < .001), TCA utilization (HR 1.43, 95%CI 1.04–1.97, *p* = .027), and atypical antidepressant usage (HR 1.52, 95%CI 1.15–2.02, *p* < .004) were associated with worse survival. On complete case analysis, SSRI use (HR 1.25, 95%CI 1.02–1.54, *p* = .035), serotonin modulator use (HR 1.54, 95%CI 1.27–1.87, *p* < .001), and TCA use (HR 1.84, 95%CI 1.21–2.80, *p* = .005) were associated with worse survival (Fig. 1, Supplementary Digital Content, Table S2). Polytherapy was similarly associated with worse overall survival (HR 1.61, 95%CI 1.31–1.98, *p* < .001) (Fig. 2). For increased robustness, in a subgroup analysis of patients with depression or anxiety, antidepressant use was associated with worse overall survival (HR 2.46, 95%CI 1.85–3.26, *p* < .001) (Supplemental Digital Content, Table S3). Further subgroup analysis within SSRI drugs were assessed due to the variation in prescribed SSRIs. Escitalopram (HR 1.33, 95%CI 1.10–1.60, *p* = .003) and citalopram (HR 1.31, 95%CI 1.01–1.70, *p* = .044) were associated with worse overall survival, while fluoxetine, paroxetine, and sertraline did not convey a survival disadvantage (Fig. 4).

**Fig. 3:**
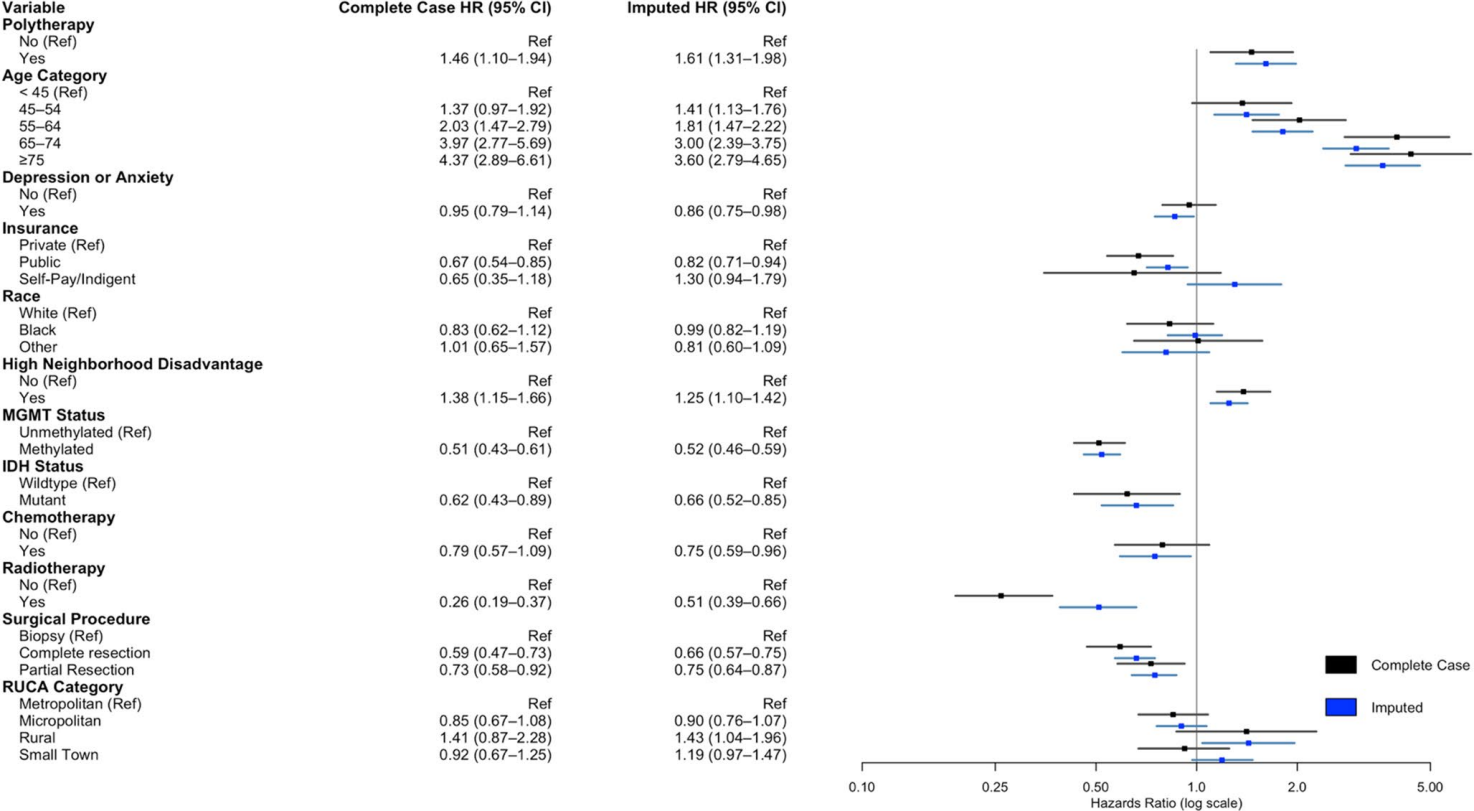
Multivariate cox regression model for impact of polytherapy on glioblastoma survival

## Discussion

**Fig. 4:**
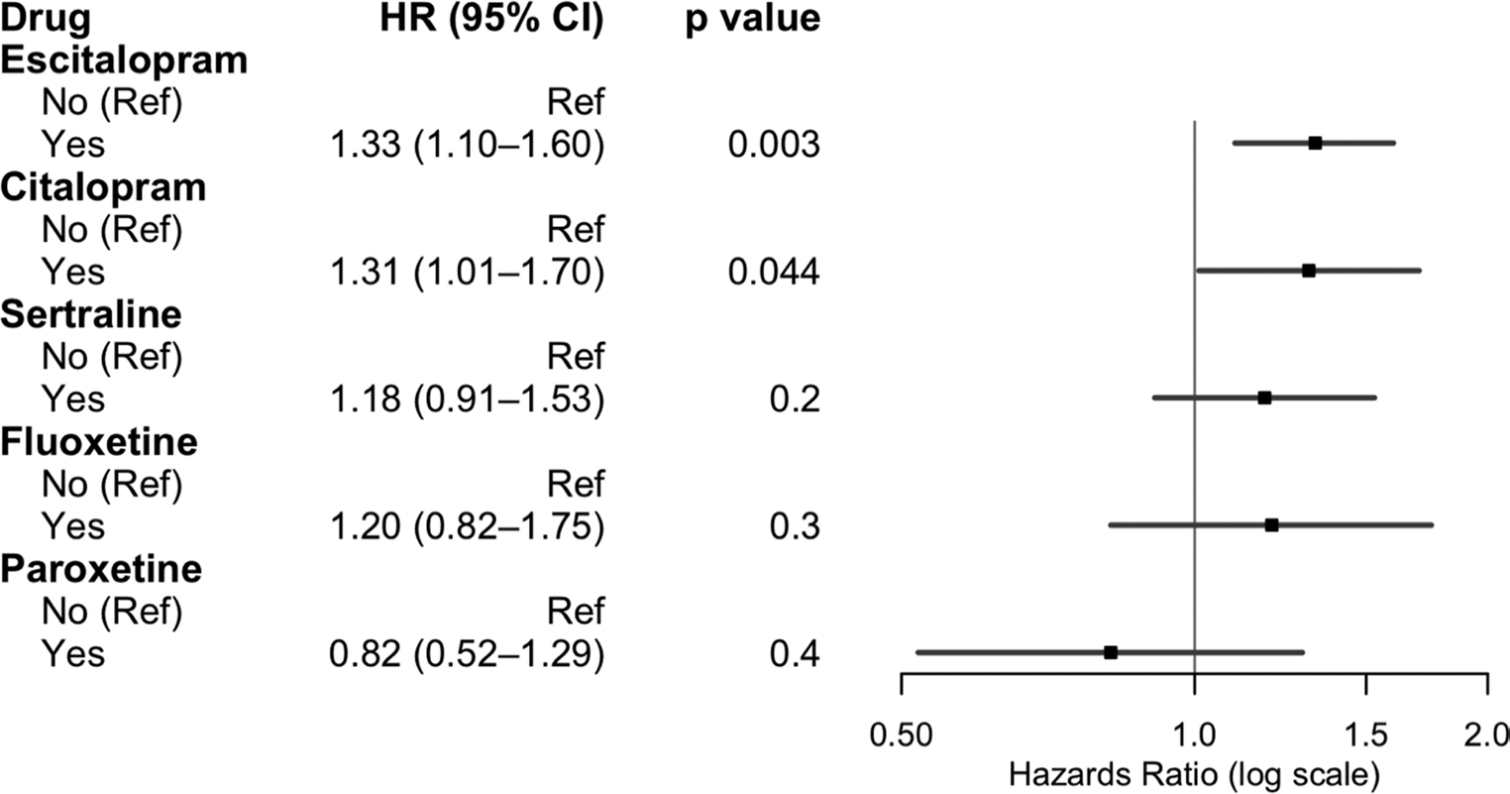
Multivariate cox regression model for impact of most used SSRIs on glioblastoma survival

Our findings suggest that utilization of antidepressants after glioblastoma diagnosis is associated with worse overall survival in patients with glioblastoma, with SSRI, serotonin modulator use, and TCA use were most strongly associated with decreased survival after adjusting for biochemical data, comorbid psychiatric conditions, treatment regimen, and other clinical and socioeconomic factors. With the disproportionally high rates of depression in glioblastoma patients, some patients may be placed on antidepressant therapy for symptomatic relief [33]. However, the effect of antidepressant therapy on survival outcomes in glioblastoma remains inconclusive [18, 19] (Fig. 4).

In our study, we find that antidepressant therapy, specifically therapy with SSRIs, serotonin modulators, and TCAs, are associated with worse survival. This is supported by several studies in literature. Gramatski et al. [34] reported antidepressant usage to not be associated with any survival improvement in a review of a registry that included 404 patients. Similarly, an analysis by Otto-Meyer et al. [17] found that no significant difference in survival between patients that had taken antidepressants. Edstrom et al. [16] demonstrated that SSRI therapy and SNRI were associated worsened survival. In an analysis of patients enrolled in clinical trials for glioblastoma, it was observed that antidepressant use during treatment for glioblastoma was associated with worsened survival [15].

This is supported by a wealth of preclinical data. Glioblastoma have been found to express serotonin receptors, of which agonism has been found to increase growth [35, 36]. Serotoninergic medications may globally increase serotonin levels and increase the known autocrine signaling loops that drive glioblastoma proliferation, though the significant heterogeneity of glioblastoma serotonin receptor expression should be noted [37]. Serotonergic medications may modulate IL-6, activating STAT3 and NF-κB to promote glioblastoma proliferation [38, 39]. Additionally, antidepressant therapy may affect the efficacy of standard chemotherapeutics utilized in glioblastoma therapy. For example, imipramine and tranylcypromine were found to temper the cytotoxic action of temozolomide by Bielecka et al. [40] under preclinical situations.

However, our results are in opposition to Caudill et al. [14] and Bi et al. [11] The mechanisms by which this may be occurring are many fold. Bi et al. [11] demonstrated that the ability of fluoxetine to inhibit sphingomyelin phosphodiesterase 1 (SMPD1), a key protein required for lipid synthesis, was a potential mechanism for the anti-glioblastoma effects of fluoxetine. There also is extensive preclinical literature highlighting these associations. Many other preclinical studies have demonstrated the ability of antidepressants to affect glioblastoma growth [40–43]. For example, studies have demonstrated the ability of fluoxetine to inhibit NF-κB signaling, inducing apoptosis in glioblastoma cells [10]. Others have demonstrated the ability of escitalopram to damage mitochondria and induce autophagy in cell models [13, 44]. Several studies demonstrate the ability of tricyclics such as impramine in inhibiting glioblastoma cells proliferation as well [9, 41, 43].

Significantly, many of these clinical studies fail to discriminate between the major classes of antidepressants, such as SSRIs, SNRIs, TCAs, and more. Furthermore, many of these studies fail to adjust for known factors for glioblastoma survival such as biomolecular data and socioeconomic characteristics. Additionally, the sample size for glioblastoma in these studies may be a limiting factor as well. Our results offer evidence that these effects persist even after controlling for these important confounders, highlighting the need to focus on translating pre-clinical results to patient outcomes.

Interestingly, we found that patients on escitalopram and citalopram had worsened survival, though this was not observed for the other SSRIs like sertraline. This may be due to lower sample sizes leading to difficulty detecting effects in the other types of SSRIs. Sertraline may exert a neuroprotective effect through its action on sigma-1 receptors, which may also account for our observations [45]. Similarly, fluoxetine has been shown to reduce MGMT expression via disruption of the NF-κB pathway, sensitizing cells to temozolomide (TMZ) in vitro and in vivo, which may account for our observations [46]. Paroxetine was also not significantly associated with worse survival. This may be due to slightly different mechanism of action of paroxetine on glioblastoma cells. Preclinical evidence has found that paroxetine induces intrinsic pathways of apoptosis in glioblastoma, which may prolong survival in some patients [47].

Polytherapy was also associated with worse survival. This may be due to similar mechanisms as previously described, with additional compounding of pro-survival effects due to polytherapy. Patients on polytherapy may also have worsened disease progression, as additions of polytherapy for depression suggests clinical states refractory to monotherapy. This is consistent with our observations that the most common polytherapy regimens are consistent with commonly prescribed add-on therapy for severe major depression [48]. This may reflect increasing disease progression and worsened state, which may be unaccounted for despite controlling for comorbid depression/anxiety in our survival models. Of note, the median treatment duration of 111 days may reflect early discontinuation due to clinical deterioration or poor adherence, which may affect outcomes and introduce bias. It is important to recognize that depression itself may represent a negative prognostic marker, though the presence of depression and/or anxiety was adjusted for in our analyses. Studies have demonstrated that patients with depression often have reduced compliance with complex therapeutic regimens which may affect outcomes [49].

These findings may also highlight an underlying interaction between antidepressant medication therapy and altered connectivity environments in glioblastoma. Recent studies have suggested that glioblastoma neural synapses are a driving force for glioblastoma growth and resistance to treatment [50, 51]. It is possible that antidepressants may modulate these networks and increase glioblastoma growth.

Our results highlight the importance of understanding the effect of pre-clinical study results in real patient populations, as clinical studies have significant heterogeneity, and findings are often not consistent with preclinical findings. Differences between in-vitro dosing and tumor micro-environment concentrations may contribute to discrepancies between preclinical and clinical data [52]. Timing of dosing and pharmacokinetic factors may differ in study settings as well. Genetic variations in drug metabolism may also further contribute to differences in results. Similarly, complex characteristics of glioblastoma microenvironments may affect drug interactions and contribute to poor generalizability from the preclinical setting to real-world data [53].

This data suggests only certain classes of antidepressants are associated with poor survival in glioblastoma when considering all relevant clinical and socioeconomic factors, supporting careful selection of medications when treating depression in glioblastoma. While proper treatment of depression and anxiety is key in improving quality of life in patients with glioblastoma, careful selection of antidepressants may be crucial, along with attention to medication adherence. Additionally, referral to psychotherapy for combined CBT (cognitive behavioral therapy) and pharmacotherapy is shown to have the highest efficacy in treating these conditions [54]. Our data suggests sertraline, paroxetine, and fluoxetine are popular SSRIs utilized clinically and may not be associated with poor outcomes. Clinicians may consider the risks and benefits of prescribing pharmacological therapies for the treatment of comorbid depression in patients with glioblastoma. Further research and higher-level evidence are necessary to better understand the impact of antidepressant therapy in glioblastoma survival.

### Limitations

Our study is limited by its retrospective, single institution design. Due to this, we may not be able to control for unknown confounders. Furthermore, our study does not consider socioeconomic status, which has been shown to potentially significantly affect glioblastoma outcomes. However, we accounted for race and rurality in our analysis. A potential limitation is the fact that poor functional status may predict increased antidepressant usage, biasing our results. However, our adjustment for baseline mental health status as well as modeling exposure as a time varying covariate should account for this to some degree. There is also potential that our review of medication records may overestimate actual usage, as compliance with medication regimen is difficult to ensure. Though we included the most common drugs given for antidepressant therapy, it is possible that there are more rare antidepressant therapies that were not included for analysis. Though there may be risk of bias due to the single institution nation of this study, our center is the primary tertiary referral center for several states in the southeastern United States, and the only NCI-designated cancer center in the state. Thus, it may be reasoned that we have an adequate sampling of the glioblastoma patients in our region. Potential interactions with other psycho-effective medications were not investigated. Revised definition of the WHO Central Nervous System (CNS) Tumor guidelines have categorized IDH mutant, Grade 4 astrocytoma as separate from glioblastoma. However, all IDH-mutant tumors were still included in this analysis to better understand the effect of antidepressant therapy and survival in high grade gliomas. We attempted to address this by controlling for biomolecular markers. There was significant missing data for IDH and MGMT marker status in the cohort, due to changes in patterns of practice prior to the 2016 WHO CNS guidelines. Thus, we were reasonably justified in assuming that data was missing in patterns that met criteria for missing-at-random (MAR), justifying the utilization of imputation methods at higher proportions of missingness [29, 55]. Furthermore, we replicated our findings in several different cohorts, further reinforcing the robustness of our findings.

## Conclusion

Utilization of SSRI, serotonin modulator use, and TCAs after glioblastoma diagnosis are associated with worse survival in patients, after adjusting for known factors with relevance to survival. Clinicians should consider the risks and benefits of prescribing antidepressants in patients with glioblastoma. Further evidence is necessary to better understand the impact of antidepressant therapy in glioblastoma survival.

## Supplementary Information

Below is the link to the electronic supplementary material.

Supplementary Material 1

## Data Availability

Data is available upon reasonable request.

## Abbreviations

ADI: Area Deprivation Index
ERS: Economic Research Service
FIPS: Federal Information Processing Standards
HRSA: Health Resources and Services Administration
SSRI: Selective Serotonin Reuptake Inhibitor
SNRI: Serotonin-Norepinephrine Reuptake Inhibitor
SMOD: Serotonin Modulator
MAOI: Monoamine Oxidase Inhibitor
TCA: Tricyclic Antidepressant
