# Supplementary material for: Survival outcomes associated with antidepressant use in glioblastoma: a cohort study
Source: J Neurooncol. 2025 Oct 27;176(1):47. doi: 10.1007/s11060-025-05288-3 (PMC12559032; doi:10.1007/s11060-025-05288-3)
Supplement: Supplementary file 1 — Supplementary Material 1 [file 11060_2025_5288_MOESM1_ESM.docx]

**SUPPLEMENTARY DIGITAL CONTENT**

**SUPPLEMENTARY METHODS**

Antidepressants were defined into 5 categories: selective serotonin reuptake inhibitors (SSRIs), serotonin/norepinephrine reuptake inhibitors (SNRIs), serotonin modulators (SMODs), tricyclic antidepressants (TCAs), and atypical antidepressants. The most common drugs for each category were selected for inclusion. Drugs in the SSRIs category were sertraline, fluvoxamine, fluoxetine, paroxetine, citalopram, and escitalopram. Drugs in the SNRIs category include venlafaxine, desvenlafaxine, duloxetine, milnacipran, levomilnacipran. Drugs in the SMODs category included nefazodone, trazodone, vilazodone, and vortioxetine. Drugs in the TCA category were amitriptyline, clomipramine, doxepin, imipramine, trimipramine, desipramine, nortriptyline, protriptyline, maprotiline, and amoxapine. Drugs in the atypical antidepressant category included bupropion, mirtazapine, agomelatine.

**Table S1.** Drug Utilization

| Drug Name | N |
| --- | --- |
| FLUOXETINE | 39 |
| PAROXETINE | 23 |
| SERTRALINE | 82 |
| CITALOPRAM | 77 |
| ESCITALOPRAM | 181 |
| FLUVOXAMINE | 1 |
| VENLAFAXINE | 41 |
| DESVENLAFAXINE | 4 |
| DULOXETINE | 48 |
| MILNACIPRAN | 0 |
| LEVOMILNACIPRAN | 0 |
| AMITRIPTYLINE | 38 |
| CLOMIPRAMINE | 1 |
| DOXEPIN | 6 |
| IMIPRAMINE | 0 |
| TRIMIPRAMINE | 0 |
| DESIPRAMINE | 0 |
| NORTRIPTYLINE | 5 |
| PROTRIPTYLINE | 0 |
| MAPROTILINE | 0 |
| AMOXAPINE | 0 |
| BUPROPION | 31 |
| MIRTAZAPINE | 42 |
| AGOMELATINE | 0 |
| REBOXETINE | 0 |
| PHENELZINE | 0 |
| TRANYLCYPROMINE | 0 |
| ISOCARBOXAZID | 0 |
| SELEGILINE | 1 |
| MOCLOBEMIDE | 0 |
| RASAGILINE | 3 |
| NEFAZODONE | 0 |
| TRAZODONE | 314 |
| VILAZODONE | 4 |
| VORTIOXETINE | 0 |

**Table S2.** Cox regression models for survival with various antidepressants

| Selective Serotonin Reuptake Inhibitors | | |  | Imputed |  |  |
| --- | --- | --- | --- | --- | --- | --- |
| **Characteristic** | **HR** | **95% CI** | **p-value** | **HR** | **95% CI** | **p-value** |
| SSRI use | 1.25 | 1.02, 1.54 | 0.035 | 1.35 | 1.16, 1.57 | <0.001 |
| Age |  |  |  |  |  |  |
| < 45 | — | — |  | — | — |  |
| 45-54 | 1.37 | 0.97, 1.93 | 0.07 | 1.4 | 1.12, 1.75 | 0.004 |
| 55-64 | 2.07 | 1.50, 2.85 | <0.001 | 1.81 | 1.47, 2.22 | <0.001 |
| 65-74 | 3.97 | 2.76, 5.69 | <0.001 | 2.92 | 2.33, 3.66 | <0.001 |
| ≥75 | 4.44 | 2.93, 6.72 | <0.001 | 3.57 | 2.77, 4.61 | <0.001 |
| Comorbid Depression or Anxiety | 0.94 | 0.77, 1.13 | 0.5 | 0.83 | 0.72, 0.96 | 0.01 |
| Insurance type | |  |  |  |  |  |
| Private | — | — |  | — | — |  |
| Public | 0.68 | 0.54, 0.86 | <0.001 | 0.82 | 0.72, 0.95 | 0.006 |
| Self-Pay/Indigent | 0.67 | 0.37, 1.23 | 0.2 | 1.31 | 0.95, 1.81 | 0.1 |
| Race |  |  |  |  |  |  |
| White | — | — |  | — | — |  |
| Black | 0.86 | 0.64, 1.15 | 0.3 | 1 | 0.83, 1.21 | >0.9 |
| Other | 1.01 | 0.65, 1.57 | >0.9 | 0.82 | 0.61, 1.10 | 0.2 |
| High Neighborhood Disadvantage | | | |  |  |  |
| FALSE | — | — |  | — | — |  |
| TRUE | 1.38 | 1.15, 1.65 | <0.001 | 1.25 | 1.10, 1.41 | <0.001 |
| MGMT Status | |  |  |  |  |  |
| Methylated | — | — |  | — | — |  |
| Unmethylated | 0.52 | 0.43, 0.62 | <0.001 | 0.53 | 0.47, 0.60 | <0.001 |
| IDH status | |  |  |  |  |  |
| IDH-Mut | — | — |  | — | — |  |
| IDH-WT | 0.61 | 0.42, 0.87 | 0.006 | 0.65 | 0.51, 0.84 | <0.001 |
| Chemotherapy | |  |  |  |  |  |
| No | — | — |  | — | — |  |
| Yes | 0.78 | 0.56, 1.08 | 0.13 | 0.77 | 0.60, 0.99 | 0.04 |
| Radiotherapy | |  |  |  |  |  |
| No | — | — |  | — | — |  |
| Yes | 0.27 | 0.19, 0.39 | <0.001 | 0.51 | 0.39, 0.67 | <0.001 |
| Extent of Resection | |  |  |  |  |  |
| Biopsy | — | — |  | — | — |  |
| Gross Total Resection | 0.58 | 0.46, 0.72 | <0.001 | 0.64 | 0.56, 0.74 | <0.001 |
| Partial Resection | 0.73 | 0.58, 0.91 | 0.006 | 0.74 | 0.63, 0.87 | <0.001 |
| RUCA |  |  |  |  |  |  |
| Metropolitan | — | — |  | — | — |  |
| Micropolitan | 0.84 | 0.66, 1.07 | 0.2 | 0.9 | 0.76, 1.06 | 0.2 |
| Rural | 1.41 | 0.87, 2.28 | 0.2 | 1.4 | 1.02, 1.92 | 0.037 |
| Small Town | 0.91 | 0.67, 1.24 | 0.6 | 1.18 | 0.96, 1.45 | 0.12 |
| Abbreviations: CI = Confidence Interval, HR = Hazard Ratio | | | | | |  |

| Serotonin/Norepinephrine Reuptake Inhibitors | | | | |  |  |
| --- | --- | --- | --- | --- | --- | --- |
| **Characteristic** | **HR** | **95% CI** | **p-value** | **HR** | **95% CI** | **p-value** |
| SNRI use | 1.25 | 0.89, 1.75 | 0.2 | 1.35 | 1.05, 1.74 | 0.02 |
| Age |  |  |  |  |  |  |
| < 45 | — | — |  | — | — |  |
| 45-54 | 1.37 | 0.97, 1.92 | 0.071 | 1.4 | 1.12, 1.75 | 0.003 |
| 55-64 | 2.06 | 1.50, 2.84 | <0.001 | 1.83 | 1.49, 2.25 | <0.001 |
| 65-74 | 4.01 | 2.80, 5.76 | <0.001 | 2.99 | 2.39, 3.74 | <0.001 |
| ≥75 | 4.39 | 2.90, 6.65 | <0.001 | 3.56 | 2.76, 4.60 | <0.001 |
| Comorbid Depression or Anxiety | 0.99 | 0.83, 1.19 | >0.9 | 0.9 | 0.79, 1.03 | 0.12 |
| Insurance type | |  |  |  |  |  |
| Private | — | — |  | — | — |  |
| Public | 0.67 | 0.53, 0.84 | <0.001 | 0.82 | 0.71, 0.95 | 0.006 |
| Self-Pay/Indigent | 0.66 | 0.36, 1.20 | 0.2 | 1.31 | 0.95, 1.81 | 0.1 |
| Race |  |  |  |  |  |  |
| White | — | — |  | — | — |  |
| Black | 0.83 | 0.62, 1.11 | 0.2 | 0.97 | 0.80, 1.17 | 0.7 |
| Other | 1 | 0.64, 1.55 | >0.9 | 0.79 | 0.59, 1.07 | 0.13 |
| High Neighborhood Disadvantage | | | |  |  |  |
| FALSE | — | — |  | — | — |  |
| TRUE | 1.36 | 1.14, 1.63 | <0.001 | 1.23 | 1.08, 1.39 | 0.002 |
| MGMT Status | |  |  |  |  |  |
| Methylated | — | — |  | — | — |  |
| Unmethylated | 0.52 | 0.43, 0.62 | <0.001 | 0.52 | 0.46, 0.59 | <0.001 |
| IDH status | |  |  |  |  |  |
| IDH-Mut | — | — |  | — | — |  |
| IDH-WT | 0.61 | 0.43, 0.87 | 0.006 | 0.65 | 0.51, 0.84 | <0.001 |
| Chemotherapy | |  |  |  |  |  |
| No | — | — |  | — | — |  |
| Yes | 0.8 | 0.58, 1.11 | 0.2 | 0.78 | 0.61, 1.01 | 0.057 |
| Radiotherapy | |  |  |  |  |  |
| No | — | — |  | — | — |  |
| Yes | 0.26 | 0.19, 0.37 | <0.001 | 0.49 | 0.37, 0.64 | <0.001 |
| Extent of Resection | |  |  |  |  |  |
| Biopsy | — | — |  | — | — |  |
| Gross Total Resection | 0.58 | 0.47, 0.72 | <0.001 | 0.65 | 0.57, 0.75 | <0.001 |
| Partial Resection | 0.72 | 0.58, 0.91 | 0.006 | 0.74 | 0.63, 0.87 | <0.001 |
| RUCA |  |  |  |  |  |  |
| Metropolitan | — | — |  | — | — |  |
| Micropolitan | 0.85 | 0.67, 1.08 | 0.2 | 0.9 | 0.76, 1.06 | 0.2 |
| Rural | 1.43 | 0.88, 2.31 | 0.15 | 1.44 | 1.05, 1.97 | 0.025 |
| Small Town | 0.92 | 0.67, 1.25 | 0.6 | 1.19 | 0.97, 1.47 | 0.094 |
| Abbreviations: CI = Confidence Interval, HR = Hazard Ratio | | | | | |  |

| Serotonin Modulator use | | |  |  |  |  |
| --- | --- | --- | --- | --- | --- | --- |
| **Characteristic** | **HR** | **95% CI** | **p-value** | **HR** | **95% CI** | **p-value** |
| Serotonin Modulator use | 1.63 | 1.42, 1.88 | <0.001 | 1.63 | 1.42, 1.88 | <0.001 |
| Age |  |  |  |  |  |  |
| < 45 | — | — |  | — | — |  |
| 45-54 | 1.36 | 1.09, 1.70 | 0.008 | 1.36 | 1.09, 1.70 | 0.008 |
| 55-64 | 1.76 | 1.43, 2.17 | <0.001 | 1.76 | 1.43, 2.17 | <0.001 |
| 65-74 | 2.98 | 2.38, 3.73 | <0.001 | 2.98 | 2.38, 3.73 | <0.001 |
| ≥75 | 3.56 | 2.76, 4.60 | <0.001 | 3.56 | 2.76, 4.60 | <0.001 |
| Comorbid Depression or Anxiety | 0.87 | 0.76, 0.99 | 0.03 | 0.87 | 0.76, 0.99 | 0.03 |
| Insurance type | |  |  |  |  |  |
| Private | — | — |  | — | — |  |
| Public | 0.8 | 0.70, 0.93 | 0.002 | 0.8 | 0.70, 0.93 | 0.002 |
| Self-Pay/Indigent | 1.23 | 0.89, 1.71 | 0.2 | 1.23 | 0.89, 1.71 | 0.2 |
| Race |  |  |  |  |  |  |
| White | — | — |  | — | — |  |
| Black | 0.96 | 0.80, 1.16 | 0.7 | 0.96 | 0.80, 1.16 | 0.7 |
| Other | 0.8 | 0.59, 1.08 | 0.2 | 0.8 | 0.59, 1.08 | 0.2 |
| High Neighborhood Disadvantage | | | |  |  |  |
| FALSE | — | — |  | — | — |  |
| TRUE | 1.24 | 1.09, 1.40 | 0.001 | 1.24 | 1.09, 1.40 | 0.001 |
| MGMT Status | |  |  |  |  |  |
| Methylated | — | — |  | — | — |  |
| Unmethylated | 0.53 | 0.47, 0.60 | <0.001 | 0.53 | 0.47, 0.60 | <0.001 |
| IDH status | |  |  |  |  |  |
| IDH-Mut | — | — |  | — | — |  |
| IDH-WT | 0.7 | 0.54, 0.89 | 0.005 | 0.7 | 0.54, 0.89 | 0.005 |
| Chemotherapy | |  |  |  |  |  |
| No | — | — |  | — | — |  |
| Yes | 0.8 | 0.62, 1.02 | 0.069 | 0.8 | 0.62, 1.02 | 0.069 |
| Radiotherapy | |  |  |  |  |  |
| No | — | — |  | — | — |  |
| Yes | 0.47 | 0.37, 0.62 | <0.001 | 0.47 | 0.37, 0.62 | <0.001 |
| Extent of Resection | |  |  |  |  |  |
| Biopsy | — | — |  | — | — |  |
| Gross Total Resection | 0.65 | 0.57, 0.75 | <0.001 | 0.65 | 0.57, 0.75 | <0.001 |
| Partial Resection | 0.72 | 0.61, 0.84 | <0.001 | 0.72 | 0.61, 0.84 | <0.001 |
| RUCA |  |  |  |  |  |  |
| Metropolitan | — | — |  | — | — |  |
| Micropolitan | 0.9 | 0.76, 1.06 | 0.2 | 0.9 | 0.76, 1.06 | 0.2 |
| Rural | 1.43 | 1.04, 1.96 | 0.026 | 1.43 | 1.04, 1.96 | 0.026 |
| Small Town | 1.22 | 0.99, 1.50 | 0.062 | 1.22 | 0.99, 1.50 | 0.062 |
| Abbreviations: CI = Confidence Interval, HR = Hazard Ratio | | | | | |  |

| Tricyclics |  |  |  |  |  |  |
| --- | --- | --- | --- | --- | --- | --- |
| **Characteristic** | **HR** | **95% CI** | **p-value** | **HR** | **95% CI** | **p-value** |
| Tricyclic Use | 1.84 | 1.21, 2.80 | 0.005 | 1.43 | 1.04, 1.97 | 0.027 |
| Age |  |  |  |  |  |  |
| < 45 | — | — |  | — | — |  |
| 45-54 | 1.34 | 0.96, 1.88 | 0.09 | 1.4 | 1.12, 1.75 | 0.004 |
| 55-64 | 2.09 | 1.52, 2.87 | <0.001 | 1.85 | 1.51, 2.28 | <0.001 |
| 65-74 | 4.02 | 2.80, 5.76 | <0.001 | 2.99 | 2.39, 3.74 | <0.001 |
| ≥75 | 4.27 | 2.83, 6.45 | <0.001 | 3.55 | 2.75, 4.58 | <0.001 |
| Comorbid Depression or Anxiety | 1.01 | 0.85, 1.20 | >0.9 | 0.93 | 0.81, 1.05 | 0.2 |
| Insurance type | |  |  |  |  |  |
| Private | — | — |  | — | — |  |
| Public | 0.69 | 0.55, 0.86 | 0.001 | 0.83 | 0.72, 0.95 | 0.008 |
| Self-Pay/Indigent | 0.68 | 0.37, 1.23 | 0.2 | 1.3 | 0.94, 1.79 | 0.12 |
| Race |  |  |  |  |  |  |
| White | — | — |  | — | — |  |
| Black | 0.78 | 0.58, 1.05 | 0.1 | 0.96 | 0.80, 1.16 | 0.7 |
| Other | 1.01 | 0.65, 1.56 | >0.9 | 0.8 | 0.59, 1.07 | 0.13 |
| High Neighborhood Disadvantage | | | |  |  |  |
| FALSE | — | — |  | — | — |  |
| TRUE | 1.34 | 1.12, 1.61 | 0.002 | 1.23 | 1.08, 1.39 | 0.001 |
| MGMT Status | |  |  |  |  |  |
| Methylated | — | — |  | — | — |  |
| Unmethylated | 0.52 | 0.43, 0.62 | <0.001 | 0.52 | 0.46, 0.59 | <0.001 |
| IDH status | |  |  |  |  |  |
| IDH-Mut | — | — |  | — | — |  |
| IDH-WT | 0.59 | 0.42, 0.85 | 0.004 | 0.65 | 0.51, 0.84 | <0.001 |
| Chemotherapy | |  |  |  |  |  |
| No | — | — |  | — | — |  |
| Yes | 0.78 | 0.56, 1.09 | 0.14 | 0.78 | 0.61, 1.01 | 0.056 |
| Radiotherapy | |  |  |  |  |  |
| No | — | — |  | — | — |  |
| Yes | 0.28 | 0.20, 0.39 | <0.001 | 0.5 | 0.38, 0.65 | <0.001 |
| Extent of Resection | |  |  |  |  |  |
| Biopsy | — | — |  | — | — |  |
| Gross Total Resection | 0.58 | 0.47, 0.72 | <0.001 | 0.64 | 0.56, 0.74 | <0.001 |
| Partial Resection | 0.73 | 0.58, 0.92 | 0.007 | 0.74 | 0.63, 0.87 | <0.001 |
| RUCA |  |  |  |  |  |  |
| Metropolitan | — | — |  | — | — |  |
| Micropolitan | 0.85 | 0.67, 1.08 | 0.2 | 0.89 | 0.76, 1.06 | 0.2 |
| Rural | 1.43 | 0.88, 2.32 | 0.14 | 1.42 | 1.04, 1.95 | 0.029 |
| Small Town | 0.92 | 0.68, 1.26 | 0.6 | 1.19 | 0.97, 1.46 | 0.1 |
| Abbreviations: CI = Confidence Interval, HR = Hazard Ratio | | | | | |  |

| Monoamine Oxidase Inhibitors | | |  |  |  |  |
| --- | --- | --- | --- | --- | --- | --- |
| **Characteristic** | **HR** | **95% CI** | **p-value** | **HR** | **95% CI** | **p-value** |
| MAOI use | 0.87 | 0.22, 3.48 | 0.8 | 0.87 | 0.22, 3.48 | 0.8 |
| Age |  |  |  |  |  |  |
| < 45 | — | — |  | — | — |  |
| 45-54 | 1.4 | 1.12, 1.75 | 0.003 | 1.4 | 1.12, 1.75 | 0.003 |
| 55-64 | 1.83 | 1.49, 2.26 | <0.001 | 1.83 | 1.49, 2.26 | <0.001 |
| 65-74 | 2.98 | 2.38, 3.73 | <0.001 | 2.98 | 2.38, 3.73 | <0.001 |
| ≥75 | 3.52 | 2.72, 4.54 | <0.001 | 3.52 | 2.72, 4.54 | <0.001 |
| Comorbid Depression or Anxiety | 0.93 | 0.82, 1.06 | 0.3 | 0.93 | 0.82, 1.06 | 0.3 |
| Insurance type | |  |  |  |  |  |
| Private | — | — |  | — | — |  |
| Public | 0.83 | 0.72, 0.95 | 0.008 | 0.83 | 0.72, 0.95 | 0.008 |
| Self-Pay/Indigent | 1.31 | 0.95, 1.80 | 0.11 | 1.31 | 0.95, 1.80 | 0.11 |
| Race |  |  |  |  |  |  |
| White | — | — |  | — | — |  |
| Black | 0.96 | 0.80, 1.16 | 0.7 | 0.96 | 0.80, 1.16 | 0.7 |
| Other | 0.79 | 0.59, 1.06 | 0.12 | 0.79 | 0.59, 1.06 | 0.12 |
| High Neighborhood Disadvantage | | | |  |  |  |
| FALSE | — | — |  | — | — |  |
| TRUE | 1.23 | 1.08, 1.40 | 0.001 | 1.23 | 1.08, 1.40 | 0.001 |
| MGMT Status | |  |  |  |  |  |
| Methylated | — | — |  | — | — |  |
| Unmethylated | 0.52 | 0.46, 0.59 | <0.001 | 0.52 | 0.46, 0.59 | <0.001 |
| IDH status | |  |  |  |  |  |
| IDH-Mut | — | — |  | — | — |  |
| IDH-WT | 0.65 | 0.51, 0.84 | <0.001 | 0.65 | 0.51, 0.84 | <0.001 |
| Chemotherapy | |  |  |  |  |  |
| No | — | — |  | — | — |  |
| Yes | 0.78 | 0.61, 1.01 | 0.056 | 0.78 | 0.61, 1.01 | 0.056 |
| Radiotherapy | |  |  |  |  |  |
| No | — | — |  | — | — |  |
| Yes | 0.49 | 0.37, 0.64 | <0.001 | 0.49 | 0.37, 0.64 | <0.001 |
| Extent of Resection | |  |  |  |  |  |
| Biopsy | — | — |  | — | — |  |
| Gross Total Resection | 0.65 | 0.56, 0.74 | <0.001 | 0.65 | 0.56, 0.74 | <0.001 |
| Partial Resection | 0.74 | 0.63, 0.87 | <0.001 | 0.74 | 0.63, 0.87 | <0.001 |
| RUCA |  |  |  |  |  |  |
| Metropolitan | — | — |  | — | — |  |
| Micropolitan | 0.89 | 0.75, 1.05 | 0.2 | 0.89 | 0.75, 1.05 | 0.2 |
| Rural | 1.41 | 1.03, 1.93 | 0.034 | 1.41 | 1.03, 1.93 | 0.034 |
| Small Town | 1.19 | 0.97, 1.46 | 0.1 | 1.19 | 0.97, 1.46 | 0.1 |
| Abbreviations: CI = Confidence Interval, HR = Hazard Ratio | | | | | |  |

| Atypical Antidepressants | | |  |  |  |  |
| --- | --- | --- | --- | --- | --- | --- |
| **Characteristic** | **HR** | **95% CI** | **p-value** | **HR** | **95% CI** | **p-value** |
| Atypical Antidepressant Use | 1.33 | 0.93, 1.90 | 0.11 | 1.52 | 1.15, 2.02 | 0.004 |
| Age |  |  |  |  |  |  |
| < 45 | — | — |  | — | — |  |
| 45-54 | 1.36 | 0.97, 1.91 | 0.075 | 1.38 | 1.10, 1.73 | 0.005 |
| 55-64 | 2.07 | 1.50, 2.84 | <0.001 | 1.82 | 1.48, 2.24 | <0.001 |
| 65-74 | 3.95 | 2.75, 5.66 | <0.001 | 2.96 | 2.36, 3.70 | <0.001 |
| ≥75 | 4.22 | 2.79, 6.39 | <0.001 | 3.46 | 2.68, 4.47 | <0.001 |
| Comorbid Depression or Anxiety | 0.99 | 0.83, 1.18 | >0.9 | 0.91 | 0.79, 1.03 | 0.13 |
| Insurance type | |  |  |  |  |  |
| Private | — | — |  | — | — |  |
| Public | 0.67 | 0.54, 0.85 | <0.001 | 0.82 | 0.71, 0.94 | 0.005 |
| Self-Pay/Indigent | 0.64 | 0.35, 1.17 | 0.2 | 1.26 | 0.91, 1.75 | 0.2 |
| Race |  |  |  |  |  |  |
| White | — | — |  | — | — |  |
| Black | 0.81 | 0.61, 1.09 | 0.2 | 0.96 | 0.80, 1.16 | 0.7 |
| Other | 1.01 | 0.65, 1.56 | >0.9 | 0.8 | 0.59, 1.08 | 0.15 |
| High Neighborhood Disadvantage | | | |  |  |  |
| FALSE | — | — |  | — | — |  |
| TRUE | 1.36 | 1.13, 1.63 | <0.001 | 1.22 | 1.07, 1.38 | 0.002 |
| MGMT Status | |  |  |  |  |  |
| Methylated | — | — |  | — | — |  |
| Unmethylated | 0.52 | 0.43, 0.62 | <0.001 | 0.52 | 0.46, 0.59 | <0.001 |
| IDH status | |  |  |  |  |  |
| IDH-Mut | — | — |  | — | — |  |
| IDH-WT | 0.61 | 0.42, 0.86 | 0.006 | 0.65 | 0.51, 0.84 | <0.001 |
| Chemotherapy | |  |  |  |  |  |
| No | — | — |  | — | — |  |
| Yes | 0.8 | 0.57, 1.11 | 0.2 | 0.78 | 0.60, 1.00 | 0.048 |
| Radiotherapy | |  |  |  |  |  |
| No | — | — |  | — | — |  |
| Yes | 0.26 | 0.18, 0.37 | <0.001 | 0.49 | 0.37, 0.64 | <0.001 |
| Extent of Resection | |  |  |  |  |  |
| Biopsy | — | — |  | — | — |  |
| Gross Total Resection | 0.57 | 0.46, 0.71 | <0.001 | 0.64 | 0.56, 0.74 | <0.001 |
| Partial Resection | 0.72 | 0.57, 0.91 | 0.005 | 0.74 | 0.63, 0.87 | <0.001 |
| RUCA |  |  |  |  |  |  |
| Metropolitan | — | — |  | — | — |  |
| Micropolitan | 0.85 | 0.67, 1.09 | 0.2 | 0.9 | 0.76, 1.06 | 0.2 |
| Rural | 1.45 | 0.89, 2.35 | 0.13 | 1.45 | 1.05, 1.99 | 0.022 |
| Small Town | 0.93 | 0.68, 1.27 | 0.7 | 1.21 | 0.99, 1.50 | 0.068 |
| Abbreviations: CI = Confidence Interval, HR = Hazard Ratio | | | | | |  |

| Any antidepressant USE | |  |  |  |  |  |
| --- | --- | --- | --- | --- | --- | --- |
| **Characteristic** | **HR** | **95% CI** | **p-value** | **HR** | **95% CI** | **p-value** |
| Any antidepressant use | 1.57 | 1.38, 1.78 | <0.001 | 1.57 | 1.38, 1.78 | <0.001 |
| Age |  |  |  |  |  |  |
| < 45 | — | — |  | — | — |  |
| 45-54 | 1.34 | 1.07, 1.68 | 0.01 | 1.34 | 1.07, 1.68 | 0.01 |
| 55-64 | 1.74 | 1.41, 2.14 | <0.001 | 1.74 | 1.41, 2.14 | <0.001 |
| 65-74 | 2.81 | 2.24, 3.51 | <0.001 | 2.81 | 2.24, 3.51 | <0.001 |
| ≥75 | 3.49 | 2.71, 4.51 | <0.001 | 3.49 | 2.71, 4.51 | <0.001 |
| Comorbid Depression or Anxiety | 0.77 | 0.67, 0.88 | <0.001 | 0.77 | 0.67, 0.88 | <0.001 |
| Insurance type | |  |  |  |  |  |
| Private | — | — |  | — | — |  |
| Public | 0.81 | 0.70, 0.93 | 0.003 | 0.81 | 0.70, 0.93 | 0.003 |
| Self-Pay/Indigent | 1.23 | 0.89, 1.70 | 0.2 | 1.23 | 0.89, 1.70 | 0.2 |
| Race |  |  |  |  |  |  |
| White | — | — |  | — | — |  |
| Black | 1 | 0.83, 1.20 | >0.9 | 1 | 0.83, 1.20 | >0.9 |
| Other | 0.84 | 0.62, 1.14 | 0.3 | 0.84 | 0.62, 1.14 | 0.3 |
| High Neighborhood Disadvantage | | | |  |  |  |
| FALSE | — | — |  | — | — |  |
| TRUE | 1.23 | 1.08, 1.40 | 0.001 | 1.23 | 1.08, 1.40 | 0.001 |
| MGMT Status | |  |  |  |  |  |
| Methylated | — | — |  | — | — |  |
| Unmethylated | 0.55 | 0.48, 0.62 | <0.001 | 0.55 | 0.48, 0.62 | <0.001 |
| IDH status | |  |  |  |  |  |
| IDH-Mut | — | — |  | — | — |  |
| IDH-WT | 0.66 | 0.51, 0.84 | 0.001 | 0.66 | 0.51, 0.84 | 0.001 |
| Chemotherapy | |  |  |  |  |  |
| No | — | — |  | — | — |  |
| Yes | 0.77 | 0.60, 0.99 | 0.038 | 0.77 | 0.60, 0.99 | 0.038 |
| Radiotherapy | |  |  |  |  |  |
| No | — | — |  | — | — |  |
| Yes | 0.51 | 0.39, 0.67 | <0.001 | 0.51 | 0.39, 0.67 | <0.001 |
| Extent of Resection | |  |  |  |  |  |
| Biopsy | — | — |  | — | — |  |
| Gross Total Resection | 0.63 | 0.55, 0.73 | <0.001 | 0.63 | 0.55, 0.73 | <0.001 |
| Partial Resection | 0.72 | 0.61, 0.84 | <0.001 | 0.72 | 0.61, 0.84 | <0.001 |
| RUCA |  |  |  |  |  |  |
| Metropolitan | — | — |  | — | — |  |
| Micropolitan | 0.92 | 0.77, 1.08 | 0.3 | 0.92 | 0.77, 1.08 | 0.3 |
| Rural | 1.46 | 1.06, 2.00 | 0.019 | 1.46 | 1.06, 2.00 | 0.019 |
| Small Town | 1.22 | 0.99, 1.50 | 0.061 | 1.22 | 0.99, 1.50 | 0.061 |
| Abbreviations: CI = Confidence Interval, HR = Hazard Ratio | | | | | |  |

**Table S3.** Cox Regression analysis for antidepressant use in depression/anxiety only

| Depression Only |  |  |  |
| --- | --- | --- | --- |
| **Characteristic** | **HR** | **95% CI** | **p-value** |
| Antidepressant Use |  |  |  |
| No | — | — |  |
| Yes | 2.46 | 1.85, 3.26 | <0.001 |
| Age |  |  |  |
| < 45 | — | — |  |
| 45-54 | 1.82 | 1.22, 2.71 | 0.003 |
| 55-64 | 2.01 | 1.40, 2.89 | <0.001 |
| 65-74 | 2.69 | 1.80, 4.02 | <0.001 |
| ≥75 | 4.57 | 2.70, 7.76 | <0.001 |
| Insurance Type |  |  |  |
| Private | — | — |  |
| Public | 0.82 | 0.62, 1.09 | 0.2 |
| Self-Pay/Indigent | 2.35 | 1.15, 4.82 | 0.02 |
| Race |  |  |  |
| White | — | — |  |
| Black | 1.11 | 0.79, 1.58 | 0.5 |
| Other | 0.59 | 0.22, 1.60 | 0.3 |
| High Neighborhood Disadvantage |  |  |  |
| No | — | — |  |
| Yes | 1.51 | 1.17, 1.94 | 0.001 |
| MGMT Status |  |  |  |
| Unmethylated | — | — |  |
| Methylated | 0.52 | 0.41, 0.67 | <0.001 |
| IDH Status |  |  |  |
| Wildtype | — | — |  |
| Mutant | 0.58 | 0.37, 0.89 | 0.013 |
| Chemotherapy |  |  |  |
| No | — | — |  |
| Yes | 0.62 | 0.39, 0.99 | 0.047 |
| Radiotherapy |  |  |  |
| No | — | — |  |
| Yes | 0.18 | 0.11, 0.31 | <0.001 |
| Extent of Resection |  |  |  |
| Biopsy | — | — |  |
| Gross Total Resection | 0.75 | 0.58, 0.98 | 0.036 |
| Partial Resection | 1.11 | 0.82, 1.50 | 0.5 |
| RUCA |  |  |  |
| Metropolitan | — | — |  |
| Micropolitan | 0.91 | 0.64, 1.29 | 0.6 |
| Rural | 0.91 | 0.52, 1.59 | 0.7 |
| Small Town | 1.27 | 0.89, 1.80 | 0.2 |
| Abbreviations: CI = Confidence Interval, HR = Hazard Ratio |  |  |  |


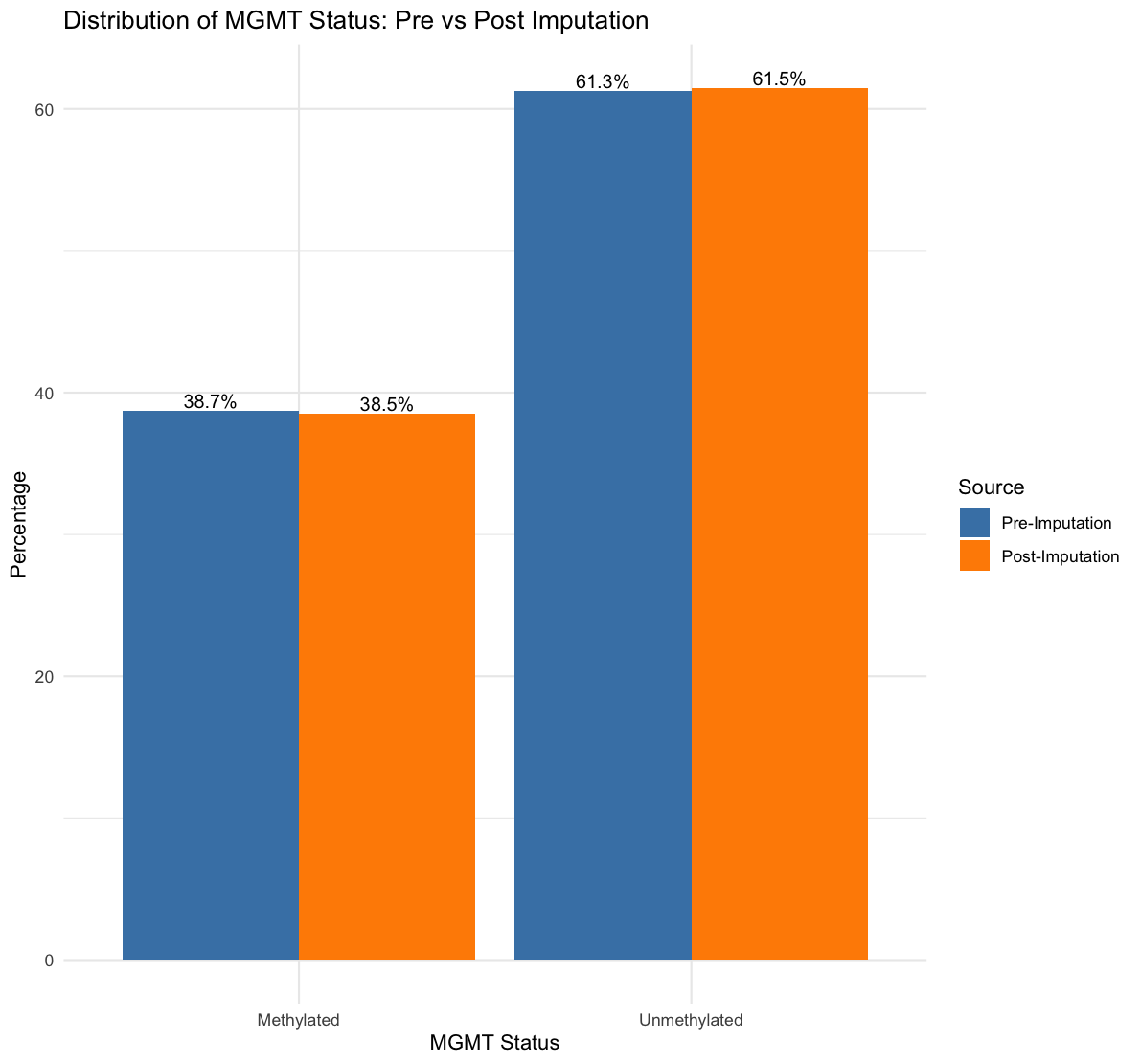

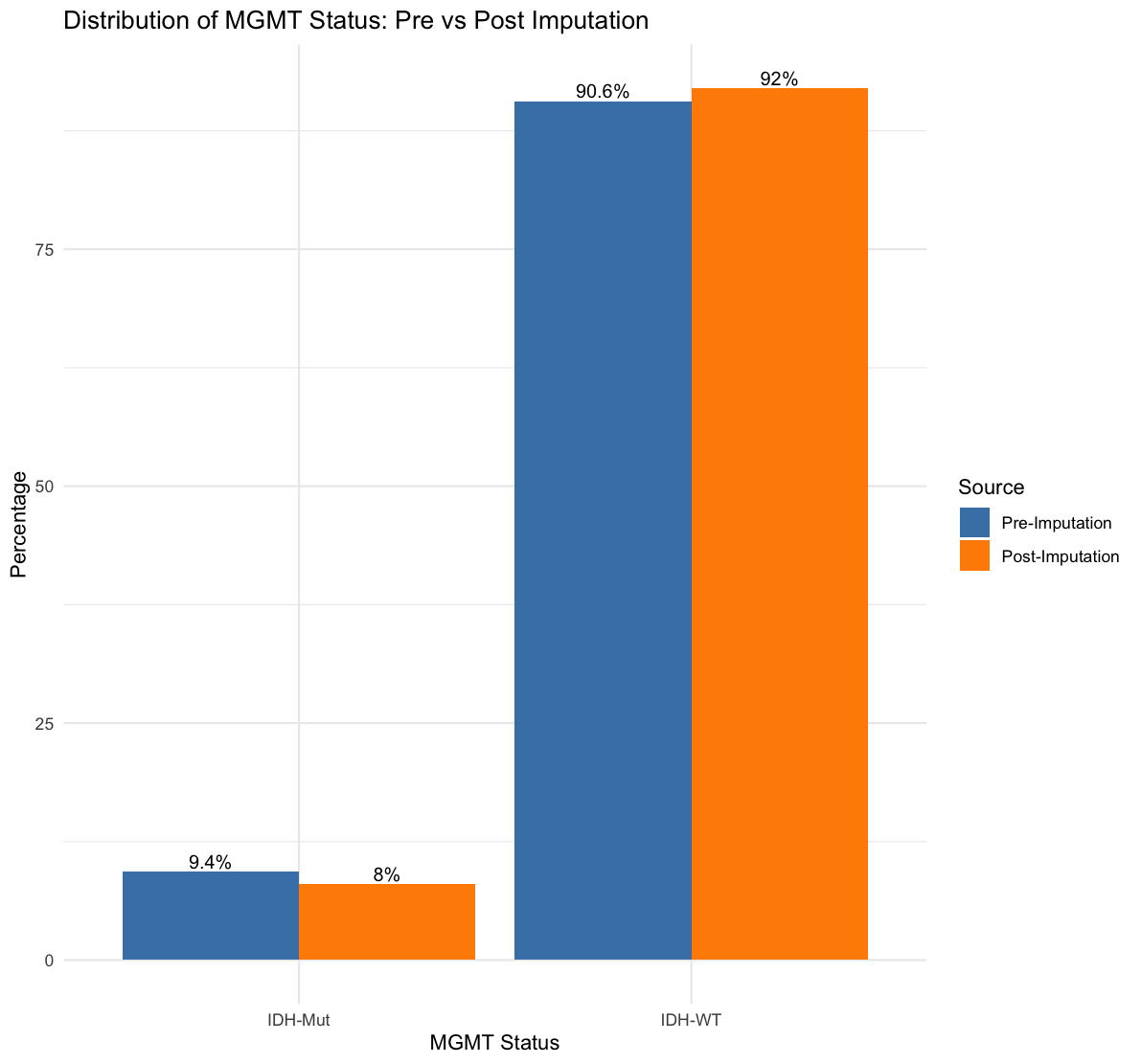


**Figure S1.** MGMT methylation and IDH status pre and post imputation


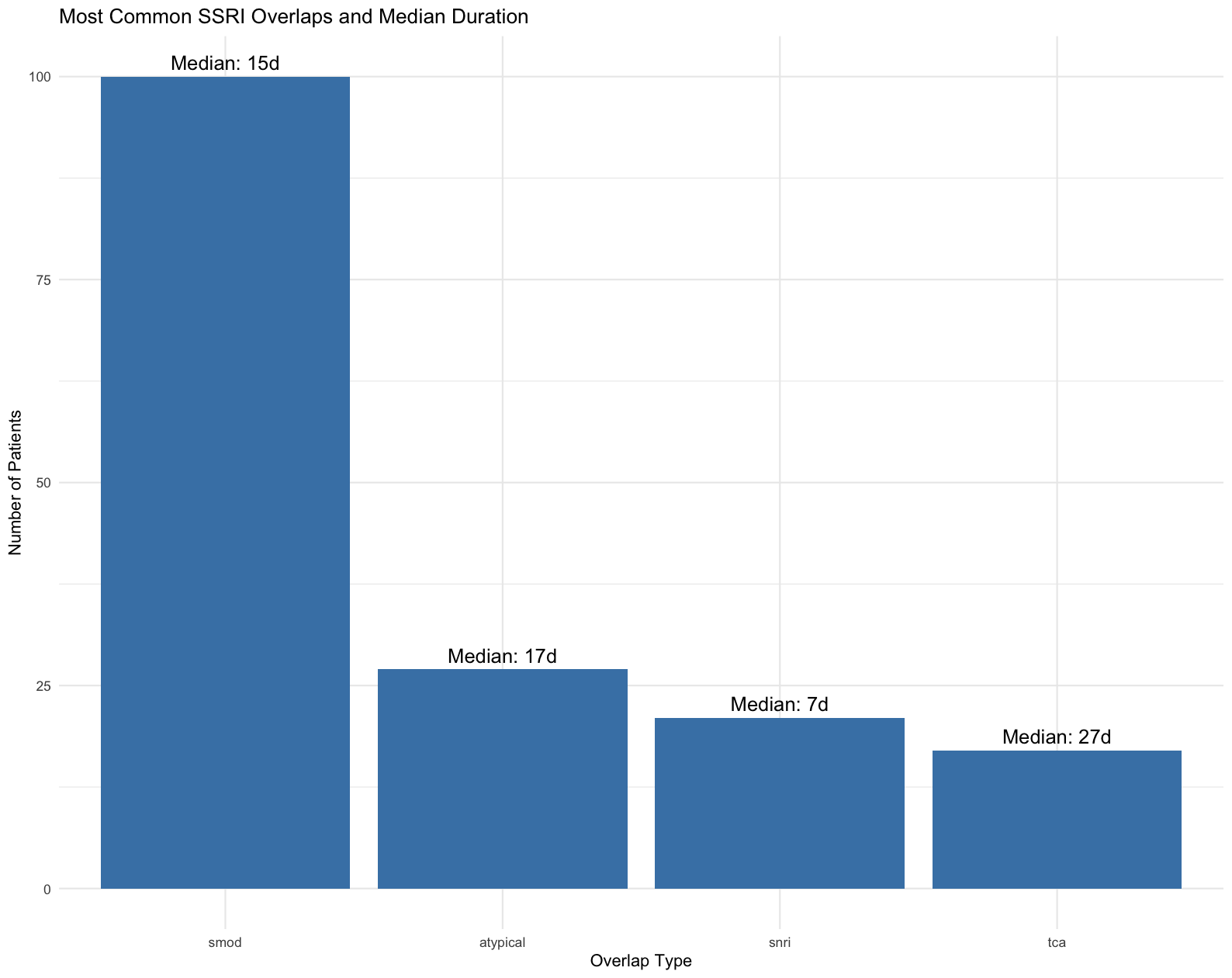


**Figure S2.** Most common forms of polytherapy
